# Exploring Associations Between Hypoxia, Serum Erythropoietin, and Cognition in Patients With Mood Disorders and Healthy Individuals

**DOI:** 10.1002/brb3.71812

**Published:** 2026-10-08

**Authors:** Barbara Emilie Rotbøl Ørum, Johanna Mariegaard Schandorff, Viktoria Damgaard, Eya‐Mist Rødgaard, Caroline Fussing Bruun, Morten Meyer, Lars Vedel Kessing, Martin Balslev Jørgensen, Kamilla Woznica Miskowiak

**Affiliations:** ^1^ Neurocognition and Emotion Across Disorders of the Brain (NEAD) Centre, Psychiatric Centre Copenhagen Frederiksberg Hospital Frederiksberg Denmark; ^2^ Department of Clinical Medicine University of Copenhagen Copenhagen Denmark; ^3^ Department of Psychology University of Copenhagen Copenhagen Denmark; ^4^ Centre For Affective Disorders Research (CADIC), Psychiatric Centre Copenhagen Frederiksberg Hospital Frederiksberg Denmark; ^5^ Department of Neurobiology Research, Institute of Molecular Medicine University of Southern Denmark Odense Denmark; ^6^ Department of Neurology Odense University Hospital Odense Denmark; ^7^ BRIDGE – Brain Research Inter‐Disciplinary Guided Excellence, Department of Clinical Research University of Southern Denmark Odense Denmark

## Abstract

**Purpose:**

Moderate repeated hypoxia (MRH) has shown neuroprotective and neuroplastic effects, partly mediated by erythropoietin (EPO) via hypoxia‐inducible factors (HIFs). This study examined the effects of 3 weeks of daily 3.5‐h normobaric hypoxia (12% O_2_) versus normoxia (20% O_2_) combined with cognitive training on oxygen saturation (SpO_2_), cerebral side effects, hematology (including EPO and brain‐derived neurotrophic factor [BDNF]), and cognitive learning. We also assessed SpO_2_ associations with side effects, hematology, and learning outcomes, as well as potential clinical and demographic predictors of hypoxic response.

**Method:**

Data from 84 healthy participants (HP) and 24 participants with remitted mood disorders (MD) in the ongoing ALTIBRAIN trial (NCT06121206) were analyzed. HP were randomized to MRH or normoxia (with/without cognitive training), while MD participants were randomized to MRH with cognitive training or treatment as usual.

**Finding:**

MRH reduced SpO_2_ (97.5% ± 1.0% to 85.9% ± 2.9%, *p* < 0.001), but this effect attenuated over time (*p* < 0.001). Side effects (e.g., dizziness and headaches) were slightly higher in MRH (*p* = 0.044) but declined over time (*p* < 0.001). Reticulocyte, hemoglobin, and thrombocyte levels increased (*p* ≤ 0.029), while EPO decreased (*p* = 0.003) and BDNF remained unchanged (*p* > 0.60). MRH slightly flattened cognitive learning curves (*p* < 0.001). MD participants experienced marginally higher side effects (*p* = 0.006), but overall tolerability was good.

**Conclusion:**

MRH was safe and well‐tolerated, influencing SpO_2_, hematology, and learning curves. If future research confirms cognitive and neuroplastic benefits, MRH with cognitive training could be a promising treatment for cognitive impairment.

## Graphical abstract

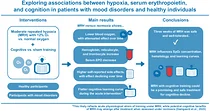

## 1 Introduction

The vital role of oxygen in life and the harms of oxygen deprivation are well‐known. It is therefore an evolutionary advantage that intermittent hypoxic conditions prompt *immediate* defense mechanisms in the organism, like increases in cardiac output, ventilation, and, with a delay of several days, increased erythrocyte production to enhance blood oxygen‐carrying capacity (Michiels, 2004; Palmer, 2010). At a cellular level, many of these mechanisms have been unraveled only in the past few decades. In 2019, William G. Kaelin Jr., Sir Peter J. Ratcliffe, and Gregg L. Semenza received a joint Nobel Prize in Physiology or Medicine for their extensive research on how cells sense and adapt to variations in oxygen availability (Zhang et al., 2019). In 1995, Semenza and Wang identified hypoxia‐inducible factor 1 (HIF‐1), a transcription factor that regulates oxygen‐dependent responses (Wang and Semenza, 1995). Under normoxic conditions, activation of HIF‐1 is inhibited (Kaelin and Ratcliffe, 2008; Lee et al., 2020; Semenza, 2012), while hypoxia stabilizes the oxygen‐sensitive subunit HIF‐1α, activating transcription of HIF‐1 genes. HIF‐1 targets genes involved in oxygen transport, including erythropoietin (EPO), vascularization, and anaerobic metabolism (Semenza, 2002; Wenger, 2002). Brain‐derived neurotrophic factor (BDNF), a neuroplasticity biomarker, has a more complex relationship with HIFs and hypoxia; however, many animal studies report upregulation of BDNF following intermittent hypoxia (Damgaard et al., 2023).

Advancing insights into hypoxia adaptation raise the question: could stimulating this pathway benefit the brain? In somatic diseases like type 2 diabetes, insufficient HIF activation is linked to impaired glucose metabolism, while hypoxia interventions improve insulin sensitivity and promote weight loss (Catrina and Zheng, 2021; Kim et al., 2021). This aligns with the concept of *hormesis*, where exposure to low‐dose stressors yields beneficial effects (Mattson, 2008). Hypoxia has shown benefits in brain cells, with an in vitro study finding that reduced oxygen enhanced neural progenitor cell proliferation, survival, and maturation (Ross et al., 2012). Supporting this, a study in a mouse model of aging has shown that oxygen restriction increased lifespan by 50% and delayed onset of neurological debility (Rogers et al., 2023). Furthermore, a study in mice showed that imitating hypoxia by stabilizing HIF led to improved hippocampal plasticity and memory (Adamcio et al., 2010). A recent systematic review investigated the effects of moderate hypoxia ≥14 days on cognition and central nervous system (CNS) outcomes across human and animal studies (Damgaard et al., 2023). Seventy‐five percent of eight different human studies reported cognitive or neurological benefits of hypoxia. Specifically, beneficial effects were observed in interventions involving low‐to‐moderate doses of hypoxia (10%‐16% O_2_) over 0.5‐4 h applied for three or more days weekly over 2–6 weeks. However, lack of blinding and randomization, coupled with small sample sizes in these studies, highlight a need for methodologically stronger studies in this field.

While several studies found CNS benefits of moderate hypoxia, the mechanism is still unclear. However, emerging evidence suggests that EPO plays a central role. EPO, primarily produced by the kidneys for erythropoiesis, is expressed in the human temporal cortex, hippocampus, and amygdala (Marti, 2004). Astrocytes and neurons are sources of brain‐derived EPO, and its receptor (EPOR) is expressed in various brain cells, including neurons, endothelial cells, microglial cells, and astrocytes. Hypoxia increases EPO mRNA threefold in primate brains (Marti, 2004; Marti et al., 1996), and EPO is upregulated following ischemic brain injury, prompting neuronal survival (Sasaki, 2003). Interestingly, constant exposure to moderate hypoxia (12%) over three weeks enhanced learning and neuroplasticity in mice undergoing motor‐cognitive training, likely via neuronal hypoxia‐induced brain EPO upregulation (Wakhloo et al., 2020). These findings are consistent with studies demonstrating cognitive and neuroplasticity benefits of systemically administered EPO. In particular, systemically administered EPO is able to cross the blood‐brain barrier in humans, exerting both neuroprotective and neurotrophic effects (Ehrenreich et al., 2004). EPO can improve cognition in patients with mood disorders (MD) (Miskowiak et al., 2014), an effect that was accompanied by increased left hippocampal volume (Miskowiak et al., 2015). However, since EPO is a hematopoietic growth factor, it requires careful monitoring by a medic throughout treatment due to the risk of thromboembolic events, especially those with somatic comorbidities (Stohlawetz et al., 2000).

Based on emerging evidence for benefits of moderate hypoxia on cognition and neuroplasticity, we initiated a translational cross‐disciplinary study investigating the effects of moderate repeated hypoxia (MRH) exposure 3.5 h, 6 days per week over 3 weeks with and without cognitive training on neuroplasticity and cognition across humans and mice (Miskowiak et al., 2024). The regimen was selected based on the findings from our previously mentioned systematic review and preclinical study (Damgaard et al., 2023; Wakhloo et al., 2020).

The present study investigates how hypoxic conditions affect physiological and cognitive learning outcomes in both healthy and cognitively impaired participants with remitted MD in our ongoing randomized controlled trial (RCT). Specifically, we aimed to (1) examine changes in SpO_2_, cerebral side effects, various biomarkers (e.g., hemoglobin, hematocrit, BDNF, and EPO), and cognitive learning during the 3‐week MRH intervention. Additionally, we aimed to (2) explore associations between SpO_2_ during MRH and side effects, hematology, and cognitive learning outcomes in healthy participants (HP) and participants with MD, and to (3) determine potential predictive roles of baseline characteristics, such as age, sex, and mental health status, on individual responses to MRH.

## 2 Methods

### 2.1 Study Design and Procedure

This study pooled data from our ongoing RCT investigating the effects of daily MRH combined with cognitive training over a three‐week period (Miskowiak et al., 2024). The trial comprises two sub‐studies supported by the European Research Council (ERC) (acronym ALTIBRAIN; grant no. 101043416): A basic scientific study (sub‐study 1) in 120 healthy humans that aims to determine the causal mechanisms of changes in neuroplasticity and cognitive function following MRH combined with cognitive training. Further, a clinical study (sub‐study 2) in 60 cognitively impaired patients with MD aims to clarify whether targeting these mechanisms can translate into enduring cognitive benefits. Sub‐study 1 employs a randomized, double‐blind, controlled design with four intervention groups: (1) normobaric hypoxia (12% O_2_) in combination with cognitive training, (2) normobaric hypoxia without cognitive training, (3) normoxia with cognitive training (20% O_2_), or (4) normoxia without cognitive training. Participants complete 18 sessions of 3.5 h, 6 days per week over 3 weeks. Sub‐study 2 involves a randomized, assessor‐blinded, controlled design with individuals with bipolar disorder (BD) or major depressive disorder (MDD) in partial or full remission who experience cognitive difficulties. Here, participants are randomized into two groups: (1) hypoxia with cognitive training or (2) a treatment‐as‐usual (TAU) control group. Participants in the first, active group complete 16 sessions of 3.5 h, 5 to 6 days per week for 3 weeks. Upon conclusion of participation, participants in the TAU arm are offered active treatment.

During sessions, participants follow a structured program including 3 × 40 min of cognitive training (using the validated HappyNeuron Pro; www.happyneuronpro.com), brief pauses, 1–2 restroom breaks, and 3 × 10 min of light treadmill walking. SpO_2_ and pulse rate are measured continuously with an oximeter (Shanghai Berry Electronic Tech Co., Ltd.). Blood samples are obtained at baseline, day 8, day 19, and week 8 to ensure safety and evaluate effects of altitude training on hematological parameters. Additionally, blood‐based biomarkers related to neuroplasticity (e.g., BDNF and EPO) are collected at baseline and day 19. Across both sub‐studies, efficacy of the intervention is assessed with neurocognitive tests, mood‐ and daily functioning ratings, as well as structural and functional neuroimaging magnetic resonance imaging (fMRI) and positron emission tomography (PET). The results for cognitive and neuroimaging measures are currently under consideration for publication elsewhere (for more details on the ALTIBRAIN study procedure, see the full protocol (Miskowiak et al., 2024)).

### 2.2 Participants and Recruitment

Sub‐study 1 includes HP aged 18–50 years with no current or prior psychiatric diagnosis confirmed using Schedules for Clinical Assessment in Neuropsychiatry (SCAN) (Wing et al., 1990). Sub‐study 2 includes participants aged 18–65 years, diagnosed with BD or MDD confirmed using SCAN. Participants must be in partial or full remission, evaluated using the Hamilton Depression Rating Scale ‐ 17 Items (HDRS (Hamilton, 1960)) and the Young Mania Rating Scale (YMRS (Young et al., 1978)), requiring scores of ≤14 on both scales. Patients are primarily recruited from the public Mental Health Services and psychiatrists in the Capital Region of Denmark. Participants must experience objective and/or subjective symptoms of cognitive impairment. Objective measures are assessed with the Screen for Cognitive Impairment in Psychiatry (SCIP (Gómez‐Benito et al., 2017)), while subjective complaints are assessed with the Cognitive Complaints in Bipolar Disorder Rating Assessment (COBRA (Rosa et al., 2013)). Comprehensive somatic and psychological screenings ensure adherence to inclusion and safety criteria. Sub‐studies 1 and 2 share exclusion criteria, including organic, substance–induced, or psychotic disorders (ICD‐10 diagnoses F0‐F29). Participants with neurological disorders, heart disease, diabetes, renal failure, hypertension, epilepsy, alcohol or substance abuse, thromboembolic events, or a first‐degree relative with a thromboembolic event before age 60 are excluded. Additional exclusions include pregnancy, breastfeeding, attempting pregnancy, current iron supplementation, smoking or regular nicotine use, and BMI > 30. More details on inclusion and exclusion criteria are available in the study protocol (Miskowiak et al., 2024). The trial has been approved by the Committee on Health Research Ethics in the Capital Region of Denmark (protocol number: H‐22028111) and the Danish Data Protection Agency in the Capital Region of Denmark (protocol number: P‐2022‐354). All participants are informed about the study procedures, and written informed consent is obtained.

### 2.3 Interventions

#### 2.3.1 Low versus Normal Ambient Oxygen Levels

Normobaric air with 12% or 20% O_2_ is administered in the same room by an air compressor with a safety‐approved system by AltitudeBalance (Miskowiak et al., 2024). For hypoxic conditions, participants enter the room at 16% O_2_ (corresponding to approx. 2,200 m altitude). To minimize the risk of altitude sickness, oxygen levels are gradually lowered over a 2‐h lead‐in phase during the first intervention day. If no adverse effects are observed, subsequent sessions reduce oxygen over 30 min to 12% (approx. 4,400 m). During intervention, participants are continuously monitored by unblinded research personnel. In the event of altitude sickness or other sudden medical emergencies, medical doctors of the project are available. To monitor altitude‐related cerebral side effects, participants complete the Environmental Symptoms Questionnaire (ESQ (Beidleman et al., 2007)) after each session. The ESQ consists of 11 items, assessing symptoms like dizziness, headache, and others. Each item is rated on a scale from 0 to 5, with 0 indicating no experience of the symptom and 5 indicating experience to an extreme degree. The total score reflects the overall severity, ranging from a total of 0 to 55 points.

#### 2.3.2 Cognitive Training versus Sham Training

Cognitive training is delivered on the web‐based, validated program HappyNeuron Pro (Danish version: 2024) (www.happyneuronpro.com), which builds on principles of neuroplasticity‐based learning by being intensive, neuroadaptive, engaging, and rewarding. Active cognitive training involves completing 26 cognitively challenging tasks within processing speed, attention, memory, and executive functions. Each task has 30 difficulty levels, and difficulty levels are comparable across tasks. On each training day, the highest level achieved for each task that the participant completed on that day is automatically saved. For the non‐active cognitive training condition (i.e., sham training), the same platform with similar, matched games is utilized; however, the sham training has low cognitive demands and does not produce cognitive benefits. Additional details on the cognitive training program are available in the trial protocol (Miskowiak et al., 2024).

### 2.4 Blood Samples

Routine blood samples are taken at inclusion, treatment days 8 and 19, and week 8 and immediately analyzed for hemoglobin, hematocrit, reticulocytes, and thrombocytes values. Research blood samples are taken at treatment days 1 and 19 and stored at ‐80°C until analysis. For these, serum was analyzed for EPO and BDNF using a custom‐made MSD U‐PLEX kit (#K151AMC‐2) (Mesoscale Discovery, Rockville, MD, USA) and a SECTOR Imager 6000 (Mesoscale Discovery) plate reader according to the manufacturer's instructions. Data were analyzed using MSD Discovery Workbench software. Analyses were performed in duplicate and reported as average values. The laboratory technician was blinded to sample identity.

### 2.5 Statistical Analyses

#### 2.5.1 Demographic and Clinical Comparisons

To assess the demographic match between treatment and diagnostic groups, we compared: (1) MRH versus normoxia in HP and (2) MRH in HP versus MRH in MD on age, sex, verbal IQ, years of education, completed days of intervention, and subsyndromal mood symptoms using chi‐square tests for categorical variables and independent samples *t*‐tests or Mann–Whitney *U* tests for normally distributed or non‐parametric continuous data, respectively.

For the analyses of Aims 1, 2, and 3, we constructed linear mixed effects models (LMMs) that can account for the observed variability in data structure from subject to subject and handle missing data. Across all models, the subject was included as a random intercept. We calculated and reported β‐values, including 95% confidence intervals for all main and interaction effects.

#### 2.5.2 Aim 1 – Comparisons Between MRH and Normoxia in HP

For Aim 1, i.e., investigating treatment effects of MRH on SpO_2_, side effects, blood‐based biomarkers, and cognitive learning in HP, we included treatment (MRH vs. normoxia), time (day of treatment), and their interaction as fixed effects. For these analyses, we only utilized data from HPs from sub‐study 1. Since the study is ongoing, data from 84 HP (MRH *n* = 44; normoxia *n* = 40) were included in the present analyses. These analyses were performed across the following dependent variables: (i) average SpO_2_ per treatment day (day 1 to 20 and, post hoc, day 2 to 20), (ii) ESQ total per treatment day, (iii) routine hematology (hemoglobin, hematocrit, reticulocytes, and thrombocytes) from inclusion, treatment day 8 and 19 (values in percent change compared to baseline), and (iv) EPO and BDNF serum values from treatment day 1 and 19. For HPs who received the active cognitive training only (MRH *n* = 23; normoxia *n* = 23), we repeated a similar LMM analysis with “cognitive learning per day” as dependent variable. For each participant, “cognitive learning per day” was calculated as the mean highest level achieved across all tasks performed on the given training day.

#### 2.5.3 Aim 2 – Investigation of Mechanistic Effects of MRH Across MD and HP Groups

For Aim 2, i.e., exploring mechanistic effects of SpO_2_ during MRH on side effects, hematology, and cognitive learning across HP and MD, we performed LMMs with average SpO_2_ per treatment day, time (day of treatment), and their interaction as fixed effects and subject as random intercept. For these analyses, we utilized data from the groups receiving MRH in both sub‐studies, comprising 24 participants with MD and 44 with HP. We included all variables that showed group differences between MRH and normoxia identified in Aim 1 as dependent variables in these models. For any blood values (routine hematology panel, EPO, and BDNF) with group differences between MRH and normoxia, we planned to calculate delta scores from treatment day 1 to 19 to correlate these with average SpO_2_ level across the treatment course. All analyses included age, verbal IQ, and number of treatment days as covariates, since HP and MD groups showed significant differences in these variables (Table 2).

#### 2.5.4 Aim 3 – Predictors of Hypoxic Response Across MD and HP Groups

Finally, for Aim 3, i.e., determining predictors of hypoxic response across HP and MD, we utilized the same dataset as in Aim 2 (MD *n* = 24; HP *n* = 44, all in MRH). We built LMMs that included potential predictors as fixed effects: group (HP vs. MD), age, sex, average SpO_2_ for treatment day 1, and depression symptoms before treatment. We also included verbal IQ and number of treatment days as fixed effects, since HP and MD groups showed significant differences in these variables (Table 2), and we wanted to explore the effects of these characteristics on hypoxic response. The subject was included as a random intercept. Variables with group differences between MRH and normoxia identified in Aim 1 were included as dependent variables.

We applied the two‐sided alpha level = 0.05. Given the exploratory and mechanistic nature of the study, we did not correct for multiple comparisons. All statistical analyses were conducted in R v.2026.01.0.

## 3 Results

### 3.1 Demographic and Clinical Comparisons

This study includes a total of 84 HP and 24 MD participants. Table 1 presents the demographics, intervention, and mood symptom characteristics of the HPs, with no significant differences between the MRH and normoxia groups (*ps* ≥ 0.12). The demographic, intervention, and clinical characteristics for the 24 MD and 44 HP participants who received MRH are shown in Table 2. MD and HP groups undergoing MRH significantly differed in age (*p* < 0.001) and verbal IQ (*p* = 0.03), but not sex and educational years (*ps* ≥ 0.11). Due to different requirements in the two sub‐studies, the groups differed in completed days of intervention (*p* < 0.001). For illness characteristics, the groups differed significantly in HDRS (*p* < 0.001) and YMRS (*p* = 0.04), as expected.

**TABLE 1:** Demographic and clinical characteristics in healthy participants in moderate repeated hypoxia (MRH) (12%) vs. normoxia (20%) groups (*N* = 84).

	MRH (*N = 44*)	Normoxia (*N* = 40)	*p*‐value
*Demographics*			
Sex (F/M%)	55%/45%	45%/55%	0.51
Age in years, median (IQR)	25 (5)	26 (8)	0.98
Educational years, median (IQR)	15 (4)	15 (4)	0.64
Verbal IQ, mean (SD)	109 (6)	109 (5)	0.89
*Intervention*			
Received cognitive training intervention (yes/no%)	52%/48%	57.5%/42.5%	0.79
Completed days of intervention, median (IQR)	17 (2)	17 (1)	0.13
*Mood symptoms*			
HDRS, median (IQR)	1 (1)	0 (1)	0.38
YMRS, median (IQR)	0 (1)	0 (0)	0.12

*Note*: Chi‐square for categorical variables, Mann–Whitney for non‐parametric data (median (IQR)), and independent *t*‐tests for normally distributed data (mean (SD)).

**Abbreviations**: HDRS = Hamilton Depression Rating Scale 17‐item version, IQ = intelligence quotient, IQR = Interquartile range, M = mean, MRH = Moderate repeated hypoxia. SD = standard deviation. YMRS = Young Mania Rating Scale.

**TABLE 2:** Demographic and clinical characteristics in participants with bipolar disorder (BD) or major depressive disorder (MDD) vs. healthy participants (HP) in the moderate repeated hypoxia (MRH) groups (*N* = 68).

	MD (*N* = 24)	HP (*N* = 44)	*p*‐value
*Demographics*			
Sex (F/M%)	75%/25%	55%/45%	0.16
Age in years, median (IQR)	33.5 (24.5)	25 (5)	**< 0.001**
Educational years, median (IQR)	15 (3)	15 (4)	0.11
Verbal IQ, mean (SD)	113 (6)	109 (6)	**0.03**
*Intervention period*			
Completed days of intervention	15 (1)	17 (2)	**< 0.001**
*Illness characteristics*			
HDRS, median (IQR)	5 (5)	1 (1)	**< 0.001**
YMRS, median (IQR)	1 (2)	0 (1)	**0.04**
Diagnosis (BD/MDD)	54%/46%	—	—
BD type I/II %	38%/62%	—	—
Illness duration, median (IQR)	17 (18)	—	—
Depressive episodes, median (IQR)	6.5 (12)	—	—
Hypomanic episodes, median (IQR)	0 (4)	—	—
Manic episodes, median (IQR)	0 (0)	—	—
Mixed state episodes, median (IQR)	0 (0)	—	—
Psychiatric hospitalizations, median (IQR)	0 (1)	—	—
Total episodes, median (IQR)	8.5 (11)	—	—
*Psychotropic medications*			
Any medication, no. (%)	19 (79%)	—	—
Antidepressants, no. (%)	13 (54%)	—	—
Antipsychotics, no. (%)	3 (13%)	—	—
Anticonvulsants, no. (%)	7 (29%)	—	—
Lithium, no. (%)	7 (29%)	—	—
Other psychoactive medications, no. (%)	3 (13%)	—	—

*Note*: Chi‐square for categorical variables, Mann–Whitney for non‐parametric data (median (IQR)), and independent *t*‐tests for normally distributed data (mean (SD)).

Abbreviations: BD = Bipolar disorder, HDRS = Hamilton Depression Rating Scale 17‐item version, HP = Healthy participants, IQ = intelligence quotient, IQR = Interquartile range, M = mean, MD = Mood disorder, MDD = Major depressive disorder, MRH = Moderate repeated hypoxia, SD = standard deviation, YMRS = Young Mania Rating Scale.

#### 3.1.1 Aim 1 ‐ Comparisons Between MRH and Normoxia in HPs

When examining the effect of MRH (vs. normoxia) on SpO_2_, we found a significant main effect of treatment (β = −11.8, 95% CI = −12.5 to −11.1, *p* < 0.001), with MRH displaying lower SpO_2_ levels (97.5% ± 1.0% to 85.9% ± 2.9%, *p* < 0.001) (Figure 1). There was no main effect of time or an SpO_2_*time interaction (*ps* ≥ 0.20), indicating stable SpO_2_ levels overall. As a post hoc analysis, we omitted day 1 (acclimatization day). This revealed a significant time‐by‐treatment interaction (β = 0.05, 95% CI = 0.022 to 0.082, *p* = < 0.001), driven by an increase in SpO_2_ over time in the MRH group compared to normoxia.

**FIGURE 1:**
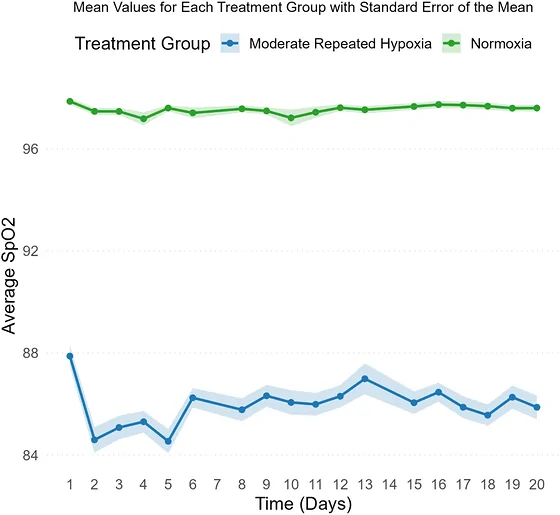
Mean of daily average SpO_2_ values (in percent) over time (days) by treatment group, with error bars representing the standard error of the mean (SEM). Blue represents MRH. Green represents normoxia.

Regarding side effects, there was a marginally significant effect of treatment on ESQ scores, with the MRH group reporting slightly more side effects than the normoxia group (β = 1.70, 95% CI = 0.07 to 3.33, *p* = 0.044) (Figure 2a). A significant time‐by‐treatment interaction (β = −0.11, 95% CI = −0.16 to −0.06, *p* < 0.001) further indicated that side effects in the MRH group decreased over time. Commonly reported symptoms included dizziness, faintness, and headaches. Figure 2b highlights the prevalence of moderate‐to‐extreme side effects (ESQ scores 3–5) which were infrequently reported (<3%) and occurred in both treatment groups.

**FIGURE 2:**
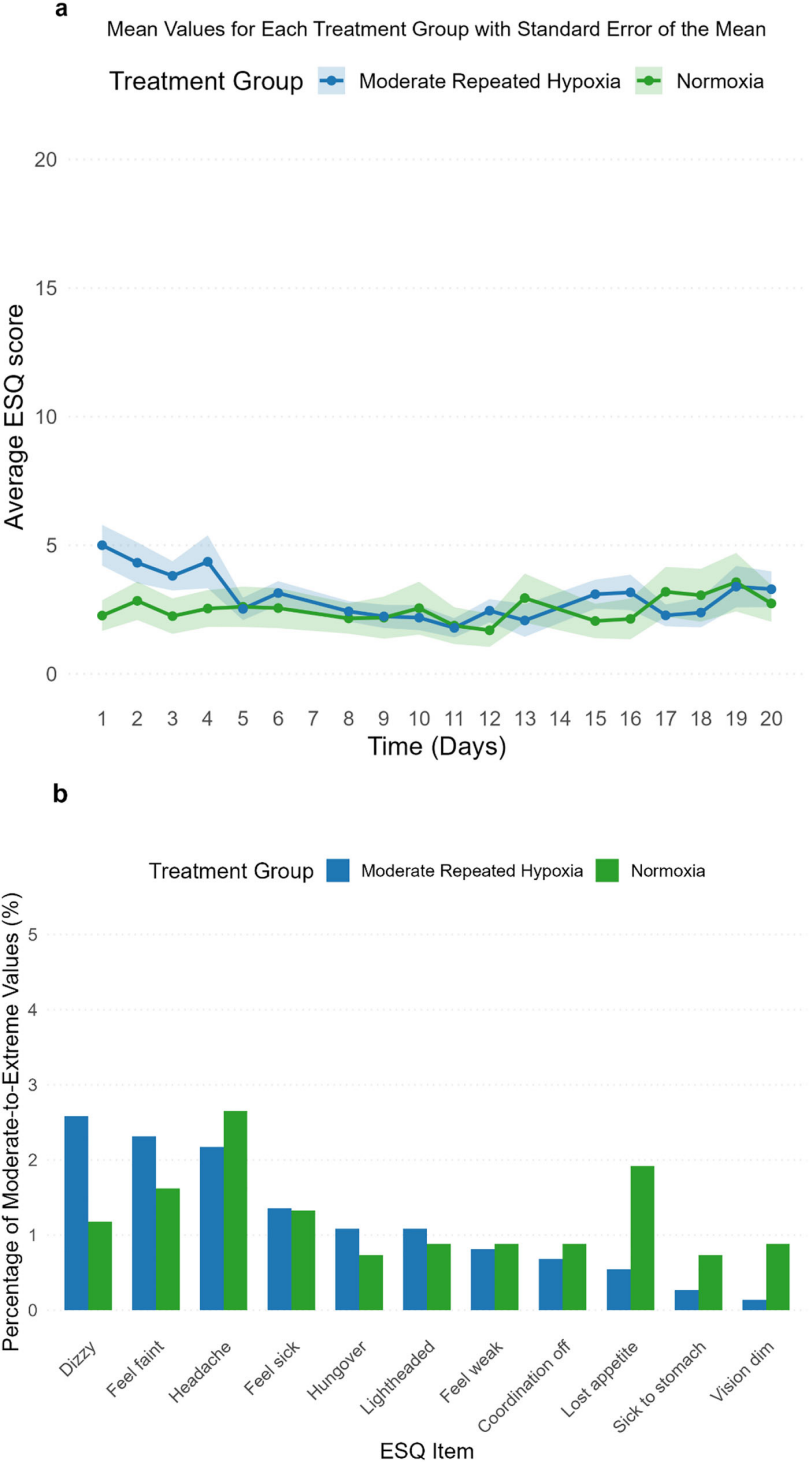
(a) Mean of daily average ESQ values (0‐55) over time (days) by treatment group, with error bars representing the standard error of the mean (SEM). Blue represents MRH. Green represents normoxia. (b) Percentage of moderate‐to‐extreme values per item in the ESQ by treatment group. Blue represents MRH. Green represents normoxia.

There was a significant time‐by‐treatment interaction for hemoglobin (β = 0.14, 95% CI = 0.02 to 0.26, *p* = 0.028) and thrombocytes (β = 0.41, 95% CI = 0.05 to 0.77, *p* = 0.029), with increases in MRH (vs. normoxia) from baseline to day 19 (Figure 3). Repeating the analysis from baseline to day 8, there was a significant time‐by‐treatment interaction for reticulocytes (β = 1.68, 95% CI = 0.36 to 3.00, *p* = 0.015), with a greater increase in MRH versus normoxia. Independent samples *t*‐tests revealed significant differences in the percent change for reticulocytes at day 8 (*p* = 0.045) and for hemoglobin (*p* = 0.025) and thrombocytes (*p* = 0.012) at day 19. No significant change was found for hematocrit (*ps* ≥ 0.10). By week 8, group differences in hemoglobin and thrombocyte levels were no longer significant (hemoglobin *ps* ≥ 0.73; thrombocyte *ps* ≥ 0.12) (Tables S1 and S2), suggesting that these changes were transient and unlikely to pose clinical risk in healthy individuals.

**FIGURE 3:**
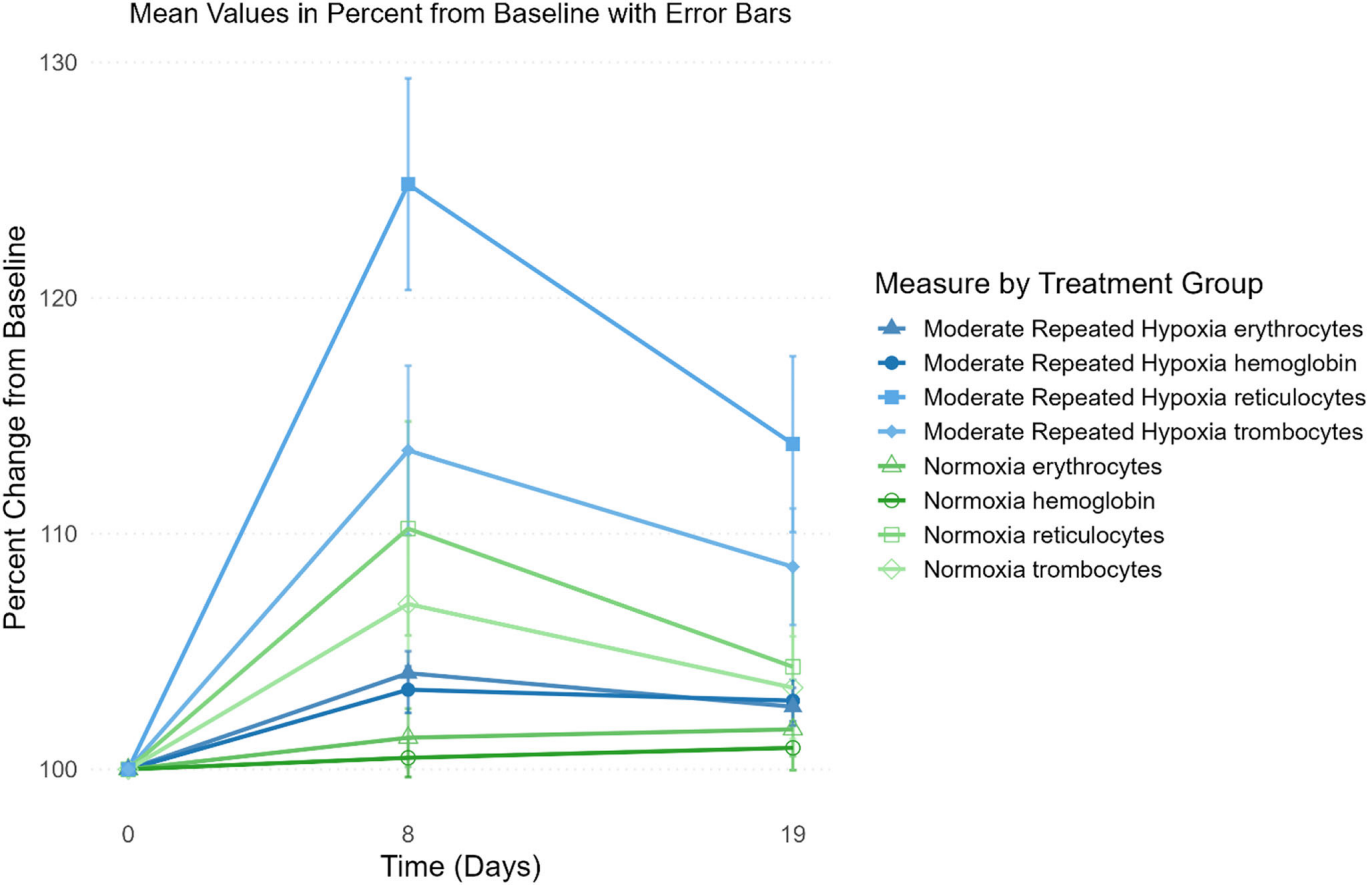
Hematology parameters (percent change from baseline) at the time points day 0, day 8, and day 19, with error bars representing the standard error of the mean (SEM). Blue represents MRH. Green represents normoxia.

Additionally, we conducted a post hoc analysis comparing these blood values from the MRH group in the current study to the active group of our previous PRETEC‐EPO trial (Macoveanu et al., 2024), where participants received EPO infusions. *T*‐tests revealed significantly greater increases in reticulocytes, hemoglobin, and hematocrit in those who received EPO versus MRH, while thrombocyte levels showed no significant difference. However, the PRETEC‐EPO trial had a longer intervention period and excluded some participants midway due to excessively elevated thrombocyte levels. Detailed results are provided in the Supplementary Material (Table S3, Figure S1).

All HP participants in both active cognitive training groups improved their cognitive performance over time independent of training under MRH or normoxia (β = 0.64, 95% CI = 0.62 to 0.67, *p* < 0.001). A significant time‐by‐treatment interaction (β = −0.12, 95% CI = −0.15 to −0.09, *p* < 0.001) revealed a slight flattening of the cognitive learning curve for the group who trained under MRH (Figure 4). However, the non‐significant main effect of treatment (*p* = 0.68) showed that average maximum levels achieved were similar across treatment groups. Independent samples *t*‐tests revealed that the slight flattening of the learning curve in the MRH versus normoxia group was driven by significant differences in the last weeks of training at the highest difficulty levels, with differences emerging on day 10 (*p* = 0.043) and persisting from days 15 to 20 (*ps* ≤ 0.045), except for day 16 (*p* = 0.13) (Table S4).

**FIGURE 4:**
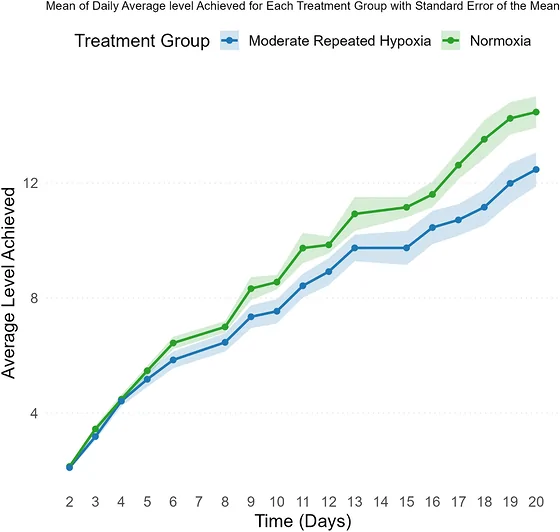
Mean of cognitive learning (daily maximum average level achieved (maximum is 30)) over time (days) by treatment group, with error bars representing the standard error of the mean (SEM). Blue represents cognitive training under MRH. Green represents cognitive training under normoxia.

There was a significant time‐by‐treatment for serum EPO levels (β = −1.36, 95% CI = ‐2.24 to ‐0.47, *p* = 0.003), with a decrease from baseline to day 19 in MRH vs. normoxia (Figure S2). Serum BDNF remained stable over time, with no significant effects of treatment, time, or their interaction (*ps* ≥ 0.60) (Figure S2). Independent samples *t*‐tests showed no baseline differences between raw serum EPO or BDNF values between treatment groups (*ps* ≥ 0.81) (Table S5).

#### 3.1.2 Aim 2 ‐ Investigation of Mechanistic Effects of MRH Across MD and HP Groups

For side effects (ESQ total score), there were no significant effects of SpO_2_, time, or their interaction (*p*s ≥ 0.50), independent of age and verbal IQ. However, there was a significant main effect of number of treatment days on ESQ total scores (β = 1.28, 95% CI = −1.93 to −0.64, *p* < 0.001), indicating that those with fewer completed treatment days also exhibited higher ESQ scores. A post hoc analysis omitting day 1 (acclimatization day), revealed a significant main effect of SpO_2_ (β = −0.17, 95% CI = ‐0.33 to ‐0.02, *p* = 0.029), in addition to the main effect of number of treatment days, suggesting higher SpO_2_ was associated with fewer side effects from day 2 and onwards. There was also a significant main effect of SpO_2_ on cognitive learning (i.e., mean highest achieved level across tasks during training) (β = 0.05, 95% CI = 0.001 to 0.10, *p* = 0.03), suggesting that higher SpO_2_ was associated with better learning, in addition to a main effect of time on learning (β = 0.78, 95% CI = 0.39 to 1.14, *p* < 0.001), but no interaction between SpO_2_*time (*p* = 0.16). For hemoglobin, thrombocytes, and EPO blood values, there was no correlation between delta scores from day 1 to day 19 and average SpO_2_ during treatment (*ps* > 0.49).

#### 3.1.3 Aim 3 ‐ Predictors of Hypoxic Response Across MD and HP Groups

Higher SpO_2_ on day 1 significantly predicted higher SpO_2_ across the three‐week treatment course (β = 0.70, 95% CI = 0.55 to 0.84, *p* < 0.001). In addition, there was a significant main effect of sex (β = 1.16, 95% CI = 0.38 to 1.94, *p* = 0.007), with males (mean = 85.0, SD = 3.11) having lower SpO_2_ than females (mean = 86.4, SD = 3.18). More completed treatment days were also significantly associated with higher SpO_2_ across the treatment course (β = 0.40, 95% CI = 0.03 to 0.76, *p* = 0.04). Group (MD vs. HP), age, verbal IQ, and baseline depression scores were not significant predictors of SpO_2_ levels (Table S6).

Side effects were mild overall. MD diagnosis predicted a higher level of side effects (ESQ) than HP (β = 3.59, 95% CI = 1.20 to 5.98, *p* = 0.006). In addition, younger age predicted a higher level of side effects measured by the ESQ (β = −0.12, 95% CI = −0.21 to −0.03, *p* = 0.02). Sex, verbal IQ, day 1 SpO_2_, and baseline depression scores were not significant predictors of ESQ. Group (MD vs. HP), sex, age, day 1 SPO_2_, number of treatment days, and baseline depression scores did not predict change in any blood‐based parameters (*ps* ≥ .12), but higher verbal IQ was associated with an increase in thrombocyte levels (β = 0.49, 95% CI = 0.05 to 0.92, *p* = 0.04). Finally, for the subset of participants undergoing active cognitive training under MRH (HP *n* = 23; MD *n* = 24), there were no significant predictors of cognitive learning, although there was a trend towards higher baseline depressive symptoms and greater cognitive learning (β = 0.05, *p* = 0.054).

## 4 Discussion

This is the first study to investigate the effects of MRH versus normoxia on SpO_2_, hematological parameters, and serum EPO and BDNF across HP and cognitively impaired participants with MD. As expected, MRH lowered SpO_2_ levels during intervention, with a slight but significant increase over time. Initially, MRH participants reported more side effects than normoxia, but the difference diminished. Notably, “moderate‐to‐extreme” side effects were reported in less than 3%, with most reporting “none‐to‐slight.” MRH caused slight but significant hemoglobin and thrombocyte increases, normalizing by week 8. Reticulocytes rose by day 8 but normalized post‐treatment. There was a slight flattening of the learning curve from those who performed cognitive training under MRH compared to normoxia. Higher SpO_2_ was associated with fewer side effects. Men exhibited lower SpO_2_ than women. Finally, younger age and MD diagnosis predicted more side effects.

As expected, MRH groups exhibited lower SpO_2_ than normoxia, but SpO_2_ increased significantly during treatment, which suggest a rightward shift in the oxygen‐dissociation curve, enhancing oxygen‐carrying capacity. Similar increases at high altitude have been observed in multiple studies. One study linked this improvement to enhanced lung diffusion, membrane diffusion, and alveolar volume (Agostoni et al., 2011). Such adaptations may represent advantageous evolutionary responses during conditions with low oxygen saturation. However, in the present experimental context, it may also attenuate the hypoxic stimulus and thereby limit the intended biological response, for example, inhibit further EPO production. Nevertheless, we still determined a substantial difference in end‐of‐treatment SpO_2_ between the MRH and normoxia groups. Additionally, hemoglobin l increased, aligning with our finding of elevated hemoglobin levels by day 19—the penultimate day of treatment. These hematological adaptations could be attributable to hypoxia‐induced HIF upregulation, activating genes responsible for physiological adaptation. HIFs peak approximately one hour after hypoxia exposure, suggesting that the 3.5‐h daily sessions likely induce peak HIF stimulation (BelAiba et al., 2007). This, in turn, upregulates EPO, thereby stimulating reticulocyte and hemoglobin production (Wenger, 2002). Additionally, our results confirm that men exhibit lower SpO_2_ at both sea level and altitude (Levental et al., 2018; Nishimura et al., 2020).

Contrary to our hypothesis, the MRH group exhibited a *decrease* in EPO during treatment compared to normoxia. A possible explanation is circulating EPO kinetics. Specifically, serum EPO concentrations peak after 19–39 h when residing at altitude 4,359 m (Abbrecht and Littell, 1972). In the current study, participants were exposed to only 3.5‐h of hypoxia daily, likely preventing peak EPO levels. Timing of blood sampling likely also influenced results, as fasting requirements meant that blood systematically was sampled in the morning before hypoxia exposure, approximately 12–20 h after the last session. Given endogenous EPO's half‐life of 5.2 h (Eckardt et al., 1989), this delay, combined with EPO not reaching peak levels, likely explains the absence of elevated EPO levels. In keeping with this, a human altitude study found that serum EPO levels returned to normal 12 h following descent from a one‐week 4,350 m altitude stay (Robach et al., 2004). Comparing *repeated normobaric* hypoxia to *continuous hypobaric* hypoxia is challenging, as the kinetics may differ. Nonetheless, the rise of reticulocytes at day 8 and subsequent hemoglobin increase suggest an increase in early EPO, given its central role in stimulating erythropoiesis (Pérez‐Ruixo et al., 2008). Earlier blood sampling might have captured a rise. The relative decrease of EPO by day 19 may reflect hormonal feedback mechanisms, where initial upregulation of EPO stimulates reticulocyte and hemoglobin production, subsequently triggering negative feedback suppressing further EPO synthesis, leading to its decline (Koulnis et al., 2014; Velan et al., 2020).

Our previous study demonstrated that an 8‐week treatment with weekly exogenous EPO (40,000 IU) improved cognitive processing speed in BD, although there was no effect on the primary outcome measure, verbal memory (Miskowiak et al., 2014). Building on this, ALTIBRAIN aimed to determine whether MRH could provide similar benefits via endogenous EPO stimulation while avoiding potential side effects of EPO treatment (Miskowiak et al., 2024). In a post hoc analysis, we directly compared the hematological parameter outcomes with that of our former 12‐week EPO trial (Macoveanu et al., 2024). In contrast to this treatment, MRH did not increase hematocrit (i.e., erythrocytes), meaning no participants required clinical intervention (i.e., bloodletting due to safety concerns), as previously reported in the EPO trial (Miskowiak et al., 2014). Direct comparison further showed that hemoglobin increases were less pronounced with MRH than weekly EPO (40,000 IU) treatment over three weeks (see Supplementary materials). Notably, MRH showed transient increases in thrombocyte levels followed by normalization at follow‐up, whereas the PRETEC‐EPO trial observed a delayed increase in thrombocyte levels, necessitating mid‐trial exclusion of some participants due to excessive increases, which are associated with increased risk of adverse events. Although these findings are interesting, such direct comparisons between the two studies should be interpreted with caution, as they differ substantially in study design, intervention (hypoxia vs. EPO infusions), study population, and intervention duration.

Contrary to our hypothesis, serum BDNF levels did not increase in either the MRH or normoxia groups. Several explanations may account for this finding. First, the intervention may simply not have produced the expected changes in brain BDNF levels. Alternatively, changes in brain BDNF may have occurred without being reflected in circulating BDNF levels. Although several studies have reported BDNF increases following hypoxia, most of these findings are based on invasive assessments that are not possible in humans, for example, sampling CNS tissue (Damgaard et al., 2023). While BDNF is produced both in the CNS and peripheral tissues, there is evidence suggesting that circulating BDNF does not consistently reflect brain BDNF levels (Béjot et al., 2011). Consequently, MRH‐induced changes in brain BDNF may have gone undetected when assessed through serum measurements alone. Finally, it is also possible that our study was underpowered to detect a statistically significant change in BDNF, since we only included a subsample of participants. The present null finding should therefore be considered preliminary until results from the complete sample are available.

Regarding side effects (ESQ scores), the MRH group reported slightly higher levels than normoxia. However, this difference diminished over time. Similar symptoms in both groups suggest a potential nocebo effect. ‘Moderate‐to‐extreme’ side effects were reported in less than 3% across all 11 ESQ items, indicating overall tolerability. MD diagnosis and younger age predicted higher side effects in MRH. Younger people may be more anxious about or sensitive to the physiological effects of hypoxia (Petersen et al., 2019). Further, greater complaints in patients may reflect reporting style, as many reported symptoms overlap with anxiety and somatization (e.g., dizziness, nausea, blurry vision, and appetite loss) (Löwe et al., 2008). Additionally, non‐blinded MD participants may have been more prone to attribute symptoms to MRH.

The MRH group receiving cognitive training showed a slight flattening of the learning curve compared to the normoxia group receiving cognitive training, which seemed to be driven by lower achieved levels during the final weeks of cognitive training. This may be due to greater physiological strain under hypoxia, limiting learning efficiency. Depending on the overall cognitive effects at the end of the trial, this observation may be consistent with existing literature, which indicates that acute hypoxia impairs certain cognitive functions when assessed *during* exposure (Chroboczek et al., 2022; Pilmanis et al., 2016; Ramírez‐delaCruz et al., 2024), while cognitive benefits are typically observed *post‐treatment* and assessed during normoxic conditions (Damgaard et al., 2023). Similarly in sports, athletes in oxygen‐intensive sports perform worse at high altitudes (Fulco et al., 1998), where beneficial effects often are reported when returning to sea level (Brocherie et al., 2017). Notably, differences in cognitive learning emerged in week two and became more pronounced in week three. As task difficulty increased over the course of training, differences between the groups thus became more evident, possibly reflecting the additional physiological demands associated with training under hypoxic conditions.

A strength of this study is its multidisciplinary approach, integrating state‐of‐the‐art psychiatric evaluation, physiology, self‐rated, performance‐based, and blood‐based measures throughout the 3‐week intervention. The high frequency of data collection provided extensive continuous data points, enabling a detailed understanding of physiological adaptations to hypoxia and yielding a thorough investigation. Although the study includes a preliminary sample size of 84 HP and 24 participants with MD in full or partial remission from this ongoing study, the design generated a substantial volume of data across a 20‐day treatment and a 5‐week follow‐up, offering valuable mechanistic insights. However, a limitation of this study is the relatively small and incomplete sample size at the time of analysis. This reduces the generalizability of findings and limits statistical power, particularly for blood test outcomes, as these were not conducted daily, resulting in fewer data points for routine hematology parameters and especially serum EPO and BDNF that were only measured at two time points. Additionally, the relationship between serum and brain‐derived EPO and BDNF is controversial, as both are produced in the periphery and the CNS. Further, the relatively small sample of individuals with mood disorders as well as the strict exclusion criteria implemented to minimize somatic complications further reduced generalizability to clinical populations, excluding many psychiatric patients with somatic comorbidities. The study's extended timeline—spanning a 3‐week intervention and a 5‐week follow‐up—posed challenges with participant attrition, particularly in the MD sub‐study. Moreover, the lack of participant blinding in the MD sub‐study increased the risk of placebo and nocebo effects, potentially affecting outcome reliability. Lastly, the association between verbal IQ and the increase in thrombocyte levels after MRH should be interpreted cautiously, as it may represent a chance finding due to multiple testing.

In conclusion, our study yielded several novel findings. The hypoxia‐related drop in SpO_2_ was attenuated over the 3‐week MRH intervention, indicating physiological adaptation. Side effects were minimal and decreased over time. In the MRH group, reticulocyte levels increased by day 8, while hemoglobin and thrombocyte levels rose by day 19. Direct comparisons with our previous study with weekly systemic EPO infusions revealed that MRH‐induced blood changes were comparable but milder, suggesting a safer treatment option. A slight flattening of the learning curve during cognitive training in MRH may reflect physiological strain or neuroplastic adaptation. The MRH and normoxia groups both reported mild side effects, which were initially slightly greater in the MRH group but diminished over time. Overall, the intervention was safe and well‐tolerated. If future research demonstrates benefits on cognitive function and neuroplasticity, MRH could emerge as a promising, safe, and viable clinical intervention for individuals with cognitive impairment.

## Author Contributions

**Barbara Emilie Rotbøl Ørum**: writing – original draft, writing – review and editing, formal analysis, data curation, project administration. **Johanna Mariegaard Schandorff**: data curation, formal analysis, writing – review and editing, conceptualization, project administration, writing – original draft. **Viktoria Damgaard**: conceptualization, data curation, formal analysis, project administration, writing – original draft, writing – review and editing. **Eya‐Mist Rødgaard**: formal analysis, writing – review and editing. **Caroline Fussing Bruun**: project administration, writing – review and editing. **Morten Meyer**: formal analysis, writing – review and editing. **Lars Vedel Kessing**: project administration, writing – review and editing. **Martin Balslev Jørgensen**: project administration, writing – review and editing. **Kamilla Woznica Miskowiak**: conceptualization, project administration, writing – original draft, writing – review and editing.

## Funding

We would like to thank the European Research Council (ERC) for financial support of the study via an ERC grant to KWM (acronym ALTIBRAIN; grant agreement No. 101043416). Additionally, we would like to thank the Lundbeck Foundation for financial support of BØ during her internship in the NEAD Centre from August 2024 to January 2025 (grant no. R432‐2023‐94).

## Ethics Statement

All participants were informed about the study procedures, and written informed consent was obtained. The study protocol was approved by the Committee on Health Research Ethics in the Capital Region of Denmark (protocol number: H‐22028111) and by the Danish Data Protection Agency at the Capital Region of Denmark (protocol number: P‐2022‐354).

## Conflicts of Interest

B.Ø., J.M.S., V.D., E.M.R., M.B.J., M.M., and C.F.B. report no conflict of interest. KWM reports having received honoraria from Lundbeck, Gedeon Richter and Angelini in the past 3 years. LVK has within the last 3 years been a consultant for Teva and Lundbeck.

## Clinical Trial Registration

https://clinicaltrials.gov/study/NCT06121206

## Supporting information




**Supplementary Material**: brb371812‐sup‐0001‐SuppMat.docx

## Data Availability

Data is available from the corresponding author upon reasonable request.

## References

[brb371812-bib-0001] Abbrecht, P. H. , and J. K. Littell . 1972. “Plasma Erythropoietin in Men and Mice During Acclimatization to Different Altitudes.” Journal of Applied Physiology 32: 54–58. 10.1152/jappl.1972.32.1.54. 5007018

[brb371812-bib-0002] Adamcio, B. , S. Sperling , N. Hagemeyer , G. Walkinshaw , and H. Ehrenreich . 2010. “Hypoxia Inducible Factor Stabilization Leads to Lasting Improvement of Hippocampal Memory in Healthy Mice.” Behavioural Brain Research 208: 80–84. 10.1016/j.bbr.2009.11.010. 19900484

[brb371812-bib-0003] Agostoni, P. , E. R. Swenson , M. Bussotti , et al. 2011. “High‐Altitude Exposure of Three Weeks Duration Increases Lung Diffusing Capacity in Humans.” Journal of Applied Physiology 110: 1564–1571. 10.1152/japplphysiol.01167.2010. 21436463

[brb371812-bib-0004] Beidleman, B. A. , S. R. Muza , C. S. Fulco , P. B. Rock , and A. Cymerman . 2007. “Validation of a Shortened Electronic Version of the Environmental Symptoms Questionnaire.” High Altitude Medicine & Biology 8: 192–199. 10.1089/ham.2007.1016. 17824819

[brb371812-bib-0005] Béjot, Y. , C. Mossiat , M. Giroud , A. Prigent‐Tessier , and C. Marie . 2011. “Circulating and Brain BDNF Levels in Stroke Rats.” Relevance to Clinical Studies 6, no. 12: e29405. 10.1371/journal.pone.0029405 PMC3241711 22195050

[brb371812-bib-0006] BelAiba, R. S. , S. Bonello , C. Zähringer , et al. 2007. “Hypoxia Up‐Regulates Hypoxia‐Inducible Factor‐1α Transcription by Involving Phosphatidylinositol 3‐Kinase and Nuclear Factor κB in Pulmonary Artery Smooth Muscle Cells.” Molecular Biology of the Cell 18: 4691–4697. 10.1091/mbc.e07-04-0391. PMC2096613 17898080

[brb371812-bib-0007] Brocherie, F. , O. Girard , R. Faiss , and G. P. Millet . 2017. “Effects of Repeated‐Sprint Training in Hypoxia on Sea‐Level Performance: A Meta‐Analysis.” Sports Medicine 47: 1651–1660. 10.1007/s40279-017-0685-3. 28194720

[brb371812-bib-0008] Catrina, S.‐B. , and X. Zheng . 2021. “Hypoxia and Hypoxia‐Inducible Factors in Diabetes and Its Complications.” Diabetologia 64: 709–716. 10.1007/s00125-021-05380-z. PMC7940280 33496820

[brb371812-bib-0009] Chroboczek, M. , S. Kujach , M. Łuszczyk , T. Grzywacz , H. Soya , and R. Laskowski . 2022. “Acute Normobaric Hypoxia Lowers Executive Functions Among Young Men Despite Increase of BDNF Concentration.” International Journal of Environmental Research and Public Health 19: 10802. 10.3390/ijerph191710802. PMC9518314 36078520

[brb371812-bib-0010] Damgaard, V. , J. Mariegaard , J. M. Lindhardsen , H. Ehrenreich , and K. W. Miskowiak . 2023. “Neuroprotective Effects of Moderate Hypoxia: A Systematic Review.” Brain Sciences 13: 1648. 10.3390/brainsci13121648. PMC10741927 38137096

[brb371812-bib-0011] Eckardt, K. U. , U. Boutellier , A. Kurtz , M. Schopen , E. A. Koller , and C. Bauer . 1989. “Rate of Erythropoietin Formation in Humans in Response to Acute Hypobaric Hypoxia.” Journal of Applied Physiology 66: 1785–1788. 10.1152/jappl.1989.66.4.1785. 2732171

[brb371812-bib-0012] Ehrenreich, H. , D. Degner , J. Meller , et al. 2004. “Erythropoietin: a Candidate Compound for Neuroprotection in Schizophrenia.” Molecular Psychiatry 9: 42–54. 10.1038/sj.mp.4001442. 14581931

[brb371812-bib-0013] Fulco, C. S. , P. B. Rock , and A. Cymerman . 1998. “Maximal and Submaximal Exercise Performance at Altitude.” Aviation Space and Environmental Medicine 69: 793–801. 9715971

[brb371812-bib-0014] Gómez‐Benito, J. , Á. I. Berrío , G. Guilera , E. Rojo , S. Purdon , and O. Pino . 2017. “The Screen for Cognitive Impairment in Psychiatry: Proposal for a Polytomous Scoring System.” International Journal of Methods in Psychiatric Research 27: e1598. 10.1002/mpr.1598. PMC6877218 29110352

[brb371812-bib-0015] Hamilton, M. 1960. “A Rating Scale for Depression.” Journal of Neurology, Neurosurgery & Psychiatry 23: 56–62. 10.1136/jnnp.23.1.56. PMC495331 14399272

[brb371812-bib-0016] Kaelin, W. G. , and P. J. Ratcliffe . 2008. “Oxygen Sensing by Metazoans: The Central Role of the HIF Hydroxylase Pathway.” Molecular Cell 30: 393–402. 10.1016/j.molcel.2008.04.009. 18498744

[brb371812-bib-0017] Kim, S.‐W. , W.‐S. Jung , S. Chung , and H.‐Y. Park . 2021. “Exercise Intervention Under Hypoxic Condition as a New Therapeutic Paradigm for Type 2 Diabetes Mellitus: A Narrative Review.” World Journal of Diabetes 12: 331–343. 10.4239/wjd.v12.i4.331. PMC8040082 33889283

[brb371812-bib-0018] Koulnis, M. , E. Porpiglia , D. Hidalgo , and M. Socolovsky . 2014 “Erythropoiesis: From Molecular Pathways to System Properties.” In A Systems Biology Approach to Blood edited by S. J. Corey , M. Kimmel , and J. N. Leonard , 37–58. Springer. 10.1007/978-1-4939-2095-210.1007/978-1-4939-2095-2_3. 25480636

[brb371812-bib-0019] Lee, P. , N. S. Chandel , and M. C. Simon . 2020. “Cellular Adaptation to Hypoxia Through Hypoxia Inducible Factors and Beyond.” Nature Reviews Molecular Cell Biology 21: 268–283. 10.1038/s41580-020-0227-y. PMC7222024 32144406

[brb371812-bib-0020] Levental, S. , E. Picard , F. Mimouni , et al. 2018. “Sex‐Linked Difference in Blood Oxygen Saturation.” The Clinical Respiratory Journal 12: 1900–1904. 10.1111/crj.12753. 29227023

[brb371812-bib-0021] Löwe, B. , R. L. Spitzer , J. B. W. Williams , M. Mussell , D. Schellberg , and K. Kroenke . 2008. “Depression, Anxiety and Somatization in Primary Care: Syndrome Overlap and Functional Impairment.” General Hospital Psychiatry 30: 191–199. 10.1016/j.genhosppsych.2008.01.001 18433651

[brb371812-bib-0022] Macoveanu, J. , J. Z. Petersen , J. Mariegaard , et al. 2024. “Effects of Erythropoietin on Cognitive Impairment and Prefrontal Cortex Activity Across Affective Disorders: A Randomized, Double‐blinded, Placebo‐controlled Trial.” Journal of Psychopharmacology 38: 362–374. 10.1177/02698811241237869. 38519416

[brb371812-bib-0023] Marti, H. H. 2004. “Erythropoietin and the Hypoxic Brain.” Journal of Experimental Biology 207: 3233–3242. 10.1242/jeb.01049. 15299044

[brb371812-bib-0024] Marti, H. H. , R. H. Wenger , L. A. Rivas , et al. 1996. “Erythropoietin Gene Expression in human, Monkey and Murine Brain.” European Journal of Neuroscience 8: 666–676. 10.1111/j.1460-9568.1996.tb01252.x. 9081618

[brb371812-bib-0025] Mattson, M. P. 2008. “Hormesis and Disease Resistance: Activation of Cellular Stress Response Pathways.” Human & Experimental Toxicology 27: 155–162. 10.1177/0960327107083417. 18480142

[brb371812-bib-0026] Michiels, C. 2004. “Physiological and Pathological Responses to Hypoxia.” The American Journal of Pathology 164: 1875–1882. 10.1016/S0002-9440(10)63747-9. PMC1615763 15161623

[brb371812-bib-0027] Miskowiak, K. W. , V. Damgaard , J. M. Schandorff , et al. 2024. “Effects of Cognitive Training Under Hypoxia on Cognitive Proficiency and Neuroplasticity in Remitted Patients With Mood Disorders and Healthy Individuals: ALTIBRAIN Study Protocol for a Randomized Controlled Trial.” Trials 25: 648. 10.1186/s13063-024-08463-5. PMC11447976 39363230

[brb371812-bib-0028] Miskowiak, K. W. , H. Ehrenreich , E. M. Christensen , L. V. Kessing , and M. Vinberg . 2014. “Recombinant human Erythropoietin to Target Cognitive Dysfunction in Bipolar Disorder: A Double‐Blind, Randomized, Placebo‐Controlled Phase 2 Trial.” The Journal of Clinical Psychiatry 75: 1347–1355. 10.4088/JCP.13m08839. 25099079

[brb371812-bib-0029] Miskowiak, K. W. , M. Vinberg , J. Macoveanu , et al. 2015. “Effects of Erythropoietin on Hippocampal Volume and Memory in Mood Disorders.” Biological Psychiatry 78: 270–277. 10.1016/j.biopsych.2014.12.013. 25641635

[brb371812-bib-0030] Nishimura, T. , J. Ugarte , M. Ohnishi , et al. 2020. “Individual Variations and Sex Differences in Hemodynamics With Percutaneous Arterial Oxygen Saturation (SpO2) in Young Andean Highlanders in Bolivia.” Journal of physiological anthropology 39, no. 1: 31.. 10.1186/s40101-020-00240-y. PMC7542971 33028423

[brb371812-bib-0031] Palmer, B. F. 2010. “Physiology and Pathophysiology With Ascent to Altitude.” The American Journal of the Medical Sciences 340: 69–77. 10.1097/MAJ.0b013e3181d3cdbe. 20442648

[brb371812-bib-0032] Pérez‐Ruixo, J. J. , W. Krzyzanski , and J. Hing . 2008. “Pharmacodynamic Analysis of Recombinant Human Erythropoietin Effect on Reticulocyte Production Rate and Age Distribution in Healthy Subjects.” Clinical Pharmacokinetics 47: 399–415. 10.2165/00003088-200847060-00004. PMC3145321 18479174

[brb371812-bib-0033] Petersen, J. Z. , R. J. Porter , and K. W. Miskowiak . 2019. “Clinical Characteristics Associated With the Discrepancy Between Subjective and Objective Cognitive Impairment in Depression.” Journal of Affective Disorders 246: 763–774. 10.1016/j.jad.2018.12.105. 30623822

[brb371812-bib-0034] Pilmanis, A. A. , U. I. Balldin , and J. R. Fischer . 2016. “Cognition Effects of Low‐Grade Hypoxia.” Aerospace Medicine and Human Performance 87: 596–603. 10.3357/AMHP.4558.2016. 27503038

[brb371812-bib-0035] Ramírez‐delaCruz, M. , A. Bravo‐Sánchez , J. Sánchez‐Infante , P. Abián , and J. Abián‐Vicén . 2024. “Effects of Acute Hypoxic Exposure in Simulated Altitude in Healthy Adults on Cognitive Performance: A Systematic Review and Meta‐Analysis.” Biology 13: 835. 10.3390/biology13100835. PMC11504018 39452143

[brb371812-bib-0036] Robach, P. , Y. Fulla , K. R. Westerterp , and J.‐P. Richalet . 2004. “Comparative Response of EPO and Soluble Transferrin Receptor at High Altitude.” Medicine & Science in Sports & Exercise 36: 1493–1498. 10.1249/01.MSS.0000139889.56481.E0. 15354029

[brb371812-bib-0037] Rogers, R. S. , H. Wang , T. J. Durham , et al. 2023. “Hypoxia Extends Lifespan and Neurological Function in a Mouse Model of Aging.” PLoS Biology 21: e3002117. 10.1371/journal.pbio.3002117. PMC10204955 37220109

[brb371812-bib-0038] Rosa, A. R. , C. Mercadé , J. Sánchez‐Moreno , et al. 2013. “Validity and Reliability of a Rating Scale on Subjective Cognitive Deficits in Bipolar Disorder (COBRA).” Journal of Affective Disorders 150: 29–36. 10.1016/j.jad.2013.02.022. 23497792

[brb371812-bib-0039] Ross, H. H. , M. S. Sandhu , T. F. Cheung , et al. 2012. “In Vivo Intermittent Hypoxia Elicits Enhanced Expansion and Neuronal Differentiation in Cultured Neural Progenitors.” Experimental Neurology 235: 238–245. 10.1016/j.expneurol.2012.01.027. PMC4089987 22366327

[brb371812-bib-0040] Sasaki, R. 2003. “Pleiotropic Functions of Erythropoietin.” Internal Medicine 42: 142–149. 10.2169/internalmedicine.42.142. 12636232

[brb371812-bib-0041] Semenza, G. L. 2002. “Signal Transduction to Hypoxia‐Inducible Factor 1.” Biochemical Pharmacology 64: 993–998. 10.1016/S0006-2952(02)01168-1. 12213597

[brb371812-bib-0042] Semenza, G. L. 2012. “Hypoxia‐Inducible Factors in Physiology and Medicine.” Cell 148: 399–408. 10.1016/j.cell.2012.01.021. PMC3437543 22304911

[brb371812-bib-0043] Stohlawetz, P. J. , L. Dzirlo , N. Hergovich , et al. 2000. “Effects of Erythropoietin on Platelet Reactivity and Thrombopoiesis in Humans.” Blood 95: 2983–2989. 10.1182/blood.V95.9.2983.009k27_2983_2989. 10779449

[brb371812-bib-0044] Velan, B. S. , L. J. Huang , and M. J. Weiss . 2020. “Erythropoietin Regulation of Red Blood Cell Production: From Bench to Bedside and Back.” F1000Research 9: F1000FacultyRev‐1153. 10.12688/f1000research.26648.1. PMC7503180 32983414

[brb371812-bib-0045] Wakhloo, D. , F. Scharkowski , Y. Curto , et al. 2020. “Functional Hypoxia Drives Neuroplasticity and Neurogenesis via Brain Erythropoietin.” Nature Communications 11: 1313. 10.1038/s41467-020-15041-1. PMC7062779 32152318

[brb371812-bib-0046] Wang, G. L. , and G. L. Semenza . 1995. “Purification and Characterization of Hypoxia‐Inducible Factor 1.” Journal of Biological Chemistry 270: 1230–1237. 10.1074/jbc.270.3.1230. 7836384

[brb371812-bib-0047] Wenger, R. H. 2002. “Cellular Adaptation to Hypoxia: O_2_ ‐Sensing Protein Hydroxylases, Hypoxia‐Inducible Transcription Factors, and O_2_ ‐Regulated Gene Expression.” The FASEB Journal 16: 1151–1162. 10.1096/fj.01-0944rev. 12153983

[brb371812-bib-0048] Wing, J. K. , T. Babor , T. Brugha , et al. 1990. “SCAN: Schedules Fonr Clinical Assessment in Neuropsychiatry.” Archives of General Psychiatry 47: 589–593. 10.1001/archpsyc.1990.01810180089012. 2190539

[brb371812-bib-0049] Young, R. C. , J. T. Biggs , V. E. Ziegler , and D. A. Meyer . 1978. “A Rating Scale for Mania: Reliability, Validity and Sensitivity.” British Journal of Psychiatry 133: 429–435. 10.1192/bjp.133.5.429. 728692

[brb371812-bib-0050] Zhang, Q. , Q. Yan , H. Yang , and W. Wei . 2019. “Oxygen Sensing and Adaptability Won the 2019 Nobel Prize in Physiology or Medicine.” Genes & Diseases 6: 328–332. 10.1016/j.gendis.2019.10.006. PMC6889041 31832511

