# Supplementary material for: Exploring Associations Between Hypoxia, Serum Erythropoietin, and Cognition in Patients With Mood Disorders and Healthy Individuals
Source: Brain Behav. 2026 Oct 8;16(10):e71812. doi: 10.1002/brb3.71812 (PMC13650406; doi:10.1002/brb3.71812)
Supplement: Supplementary file 1 [file BRB3-16-e71812-s001.docx]

**Supplementary materials**

Post hoc we performed an analysis comparing results of blood values from the hypoxia group to the active group of our research group’s former PRETEC-EPO trial^1^. In this trial participants in the active group received EPO infusions (40,000 IU), whereas the control group received saline injections. Participants receiving EPO also experienced an increase in hematology parameters. However, we hypothesized that this increase would be greater in the EPO group compared to hypoxia group in ALTIBRAIN. We performed independent samples *t-*tests, comparing the routine hematology parameters (raw values and percent change from inclusion) for each time point between hypoxia and active group in EPO trial. To ensure a direct comparison between the two studies at similar time points, we exclusively use data from the first three weeks of the PRETEC-EPO trial. At day 19, we observed a significant difference in percent change from baseline in hemoglobin between the hypoxia and EPO groups (*p* = .02) (**Table S3**) (**Figure S1**). For hematocrit we saw a highly significant difference at day 19 for both raw values and percent change from baseline (*ps* < .001). Reticulocytes were significantly different at day 8 and day 19 for both raw values and percent change from baseline (*ps* < .001). For thrombocytes no significant differences were seen (*ps* ≥ .06), indicating that the intervention in the PRETEC-EPO trial elevated blood parameters compared to ALTIBRAIN, except for thrombocytes where no significant difference was seen. Nevertheless, these direct comparisons between the two studies should be interpreted with caution, because they differ substantially in study design, intervention (hypoxia vs. EPO infusions), study population, and intervention duration.

**Table S1.** Raw blood and percent from baseline values with standard deviation (SD) and *p*-value (*p*) at baseline, day 8, and day 19. Including group minimum and maximum values.

|  |  | **Baseline** | | | | |  | **Day 8** | | | | | | **Day 19** | | | | | | |
| --- | --- | --- | --- | --- | --- | --- | --- | --- | --- | --- | --- | --- | --- | --- | --- | --- | --- | --- | --- | --- |
|  |  | Mean (SD) | *p* | Percent | Group min value | Group max value |  | Mean (SD) | *p* | % from baseline (SD) | *p* | Group min value | Group max value |  | Mean (SD) | *p* | % from baseline (SD) | *p* | Group min value | Group max value |
| **Hemoglobin** | MRH | 8.76 (0.66) | .55 | 100 | 7.6 | 10.2 |  | 9.00 (0.76) | .75 | 103 (5.84) | .10 | 7.8 | 10.7 |  | 9.06 (0.76) | .16 | 103 (5.63) | **.025** | 7.6 | 10.4 |
|  | Normoxia | 8.84 (0.64) |  | 100 | 7.5 | 10.5 |  | 8.95 (0.68) |  | 101 (3.99) |  | 7.8 | 10.6 |  | 8.85 (0.55) |  | 101 (5.25) |  | 7.8 | 9.8 |
| **Hematocrit** | MRH | 0.42 (0.03) | .88 | 100 | 0.38 | 0.47 |  | 0.43 (0.03) | .60 | 103 (5.24) | .11 | 0.39 | 0.5 |  | 0.44 (0.03) | .14 | 103 (5.21) | .17 | 0.38 | 0.51 |
|  | Normoxia | 0.43 (0.03) |  | 100 | 0.36 | 0.48 |  | 0.43 (0.03) |  | 101 (6.02) |  | 0.38 | 0.5 |  | 0.43 (0.02) |  | 101 (6.34) |  | 0.38 | 0.47 |
| **Reticulocytes** | MRH | 13.2 (5.64) | .49 | 100 | 6 | 37 |  | 15.9 (5.51) | .34 | 124 (26.3) | **.045** | 6 | 37 |  | 14.3 (4.87) | .86 | 113 (23.2) | .12 | 8 | 37 |
|  | Normoxia | 14.1 (4.19) |  | 100 | 7 | 30 |  | 14.8 (3.19) |  | 110 (22.7) |  | 8 | 22 |  | 14.2 (3.23) |  | 104 (25.0) |  | 7 | 20 |
| **Thrombocytes** | MRH | 242 (52.5) | .50 | 100 | 149 | 374 |  | 263 (52.7) | .97 | 112 (20.0) | .12 | 166 | 407 |  | 264 (55.4) | .17 | 110 (14.9) | **.012** | 165 | 392 |
|  | Normoxia | 250 (58.0) |  | 100 | 154 | 471 |  | 264 (51.2) |  | 105 (14.8) |  | 159 | 405 |  | 249 (46.3) |  | 102 (12.5) |  | 152 | 399 |
| Note: Significant values are marked in bold. Raw values are measured in [mmol/L] for hemoglobin, [volume fraction] for hematocrit, fraction reticulocytes of erythrocytes in [*10^-3] for reticulocytes, and [*10^9/L] for thrombocytes. | | | | | | | | | | | | | | | | | | | | |

**Table S2.** Raw blood and percent from baseline values with standard deviation (SD) and *p*-value (*p*) at baseline, day 8, day 19, and week 8.

|  |  | Baseline | | |  | Day 8 | | | |  | Day 19 | | | |  | Week 8 (day 56) | | | |
| --- | --- | --- | --- | --- | --- | --- | --- | --- | --- | --- | --- | --- | --- | --- | --- | --- | --- | --- | --- |
|  |  | Mean (SD) | *p* | Percent |  | Mean (SD) | *p* | Percent from baseline (SD) | *p* |  | Mean (SD) | *p* | Percent from baseline (SD) | *p* |  | Mean (SD) | *p* | Percent from baseline (SD) | *p* |
| **Hemoglobin** | MRH | 8.76 (0.66) | .55 | 100 |  | 9.00 (0.76) | .75 | 103 (5.84) | .10 |  | 9.06 (0.76) | .16 | 103 (5.63) | **.025** |  | 8.96 (0.74) | .75 | 102 (4.81) | .73 |
|  | Normoxia | 8.84 (0.64) |  | 100 |  | 8.95 (0.68) |  | 101 (3.99) |  |  | 8.85 (0.55) |  | 101 (5.25) |  |  | 8.9 (0.56) |  | 102 (4.94) |  |
| **Thrombocytes** | MRH | 242 (52.5) | .50 | 100 |  | 263 (52.7) | .97 | 112 (20.0) | .12 |  | 264 (55.4) | .17 | 110 (14.9) | **.012** |  | 252 (52.1) | .98 | 113 (15.2) | .12 |
|  | Normoxia | 250 (58.0) |  | 100 |  | 264 (51.2) |  | 105 (14.8) |  |  | 249 (46.3) |  | 102 (12.5) |  |  | 252 (45.4) |  | 104 (15.3) |  |
| Note: Significant *p*-values are marked in bold. Raw values are measured in [mmol/L] for hemoglobin, [volume fraction] for hematocrit, fraction reticulocytes of erythrocytes in [*10^-3] for reticulocytes, and [*10^9/L] for thrombocytes. | | | | | | | | | | | | | | | | | | | |

**Table S3.** Raw blood and percent from baseline values with standard deviation (SD) and *p*-value (*p*) at baseline, day 8, and day 19. Including group minimum and maximum values.

|  |  | **Baseline** | | | | | **Day 8** | | | | | | |  | **Day 19** | | | | | | |
| --- | --- | --- | --- | --- | --- | --- | --- | --- | --- | --- | --- | --- | --- | --- | --- | --- | --- | --- | --- | --- | --- |
|  |  | Mean (SD) | *p* | Percent | Group min value | Group max value |  | Mean (SD) | *p* | Percent from baseline (SD) | *p* | Group min value | Group max value |  | Mean (SD) | *p* | Percent from baseline (SD) | *p* | Group min value | Group max value | |
| **Hemoglobin** | MRH | 8.65 (0.71) | .99 | 100 | 7.3 | 10.2 |  | 8.87 (0.77) | .41 | 103 (5.53) | .14 | 7.6 | 10.7 |  | 8.93 (0.74) | .08 | 104 (5.91) | **.02** | 7.3 | 10.4 | |
|  | EPO | 8.65 (0.68) |  | 100 | 7.2 | 10.1 |  | 8.76 (0.77) |  | 101 (5.39) |  | 7.4 | 10.6 |  | 9.18 (0.77) |  | 107 (6.56) |  | 7.6 | 11.3 | |
| **Hematocrit** | MRH | 0.42 (0.03) | .61 | 100 | 0.36 | 0.48 |  | 0.43 (0.03) | .96 | 103 (5.54) | .21 | 0.37 | 0.50 |  | 0.43 (0.03) | **<.001** | 103 (5.83) | **<.001** | 0.37 | 0.51 | |
|  | EPO | 0.42 (0.03) |  | 100 | 0.35 | 0.49 |  | 0.43 (0.04) |  | 102 (5.31) |  | 0.37 | 0.52 |  | 0.46 (0.04) |  | 108 (7.06) |  | 0.38 | 0.58 | |
| **Reticulocytes** | MRH | 12.8 (4.78) | .15 | 100 | 6 | 37 |  | 15.5 (4.63) | **<.001** | 123 (29.5) | **<.001** | 6 | 37 |  | 14.1 (4.19) | **<.001** | 114 (23.3) | **<.001** | 8 | 37 | |
|  | EPO | 13.9 (3.21) |  | 100 | 7 | 22 |  | 25.0 (7.61) |  | 184 (53.9) |  | 11 | 42 |  | 21.3 (6.27) |  | 160 (51.1) |  | 8 | 37 | |
| **Thrombocytes** | MRH | 239 (47.3) | .15 | 100 | 142 | 374 |  | 265 (47.1) | .47 | 113 (17.7) | .30 | 166 | 407 |  | 261 (51.5) | .06 | 110 (13.5) | .25 | 165 | 392 | |
|  | EPO | 253 (52.4) |  | 100 | 149 | 389 |  | 272 (59.2) |  | 109 (14.9) |  | 138 | 410 |  | 281 (59.6) |  | 113 (14.4) |  | 163 | 405 | |
| Note: Significant *p*-values are marked in bold. Raw values are measured in [mmol/L] for hemoglobin, [volume fraction] for hematocrit, fraction reticulocytes of erythrocytes in [*10^-3] for reticulocytes, and [*10^9/L] for thrombocytes. | | | | | | | | | | | | | | | | | | | | |  |

**Table S4**. Average maximum levels achieved per day per group across tasks, along with standard deviations (SD) and *p*-values (*p*).

| **Week 1** | **Day 1** | | **Day 2** | | **Day 3** | | **Day 4** | | **Day 5** | | **Day 6** | | |
| --- | --- | --- | --- | --- | --- | --- | --- | --- | --- | --- | --- | --- | --- |
|  |  |  |  |  |  |  |  |  |  |  |  |  |  |
|  | No treatment | | Mean (SD) | *p* | Mean (SD) | *p* | Mean (SD) | *p* | Mean (SD) | *p* | Mean (SD) | *p* |  |
| MRH |  |  | 2.11 (0.36) | .68 | 3.19 (0.666) | .17 | 4.42 (0.869) | .81 | 5.18 (1.19) | .33 | 5.85 (1.37) | .12 |  |
| Normoxia |  |  | 2.15 (0.29) |  | 3.45 (0.588) |  | 4.48 (0.793) |  | 5.47 (0.791) |  | 6.44 (0.979) |  |  |
| **Week** **2** | **Day 8** | | **Day 9** | | **Day 10** | | **Day 11** | | **Day 12** | | **Day 13** | |  |
|  |  |  |  |  |  |  |  |  |  |  |  |  |  |
|  | Mean (SD) | *p* | Mean (SD) | *p* | Mean (SD) | *p* | Mean (SD) | *p* | Mean (SD) | *p* | Mean (SD) | *p* |  |
| MRH | 6.46 (1.5) | .16 | 7.35 (1.85) | .09 | 7.54 (1.93) | **.043** | 8.42 (1.92) | .058 | 8.92 (2.24) | .10 | 9.74 (1.79) | .16 |  |
| Normoxia | 7.0 (0.933) |  | 8.33 (1.88) |  | 8.55 (1.17) |  | 9.74 (2.53) |  | 9.85 (1.4) |  | 10.9 (2.83) |  |  |
| **Week 3** | **Day 15** | | **Day 16** | | **Day 17** | | **Day 18** | | **Day 19** | | **Day 20** | |  |
|  |  |  |  |  |  |  |  |  |  |  |  |  |  |
|  | Mean (SD) | *p* | Mean (SD) | *p* | Mean (SD) | p | Mean (SD) | p | Mean (SD) | *p* | Mean (SD) | *p* |  |
| MRH | 9.74 (2.72) | **.045** | 10.4 (2.71) | .13 | 10.7 (2.63) | **.013** | 11.2 (2.99) | **.013** | 12.0 (3.25) | **.015** | 12.5 (2.49) | **.018** |  |
| Normoxia | 11.2 (1.64) |  | 11.6 (1.99) |  | 12.6 (2.34) |  | 13.5 (3.18) |  | 14.2 (2.69) |  | 14.5 (2.58) |  |  |
| Note: Maximum possible level is 30. Significant *p*-values are marked in bold. Days 7 and 14 are not shown, since these were mandatory rest days, and no participant thus trained on these days. The final intervention day was day 20. | | | | | | | | | | | | | |

**Table S5.** Raw serum and percent from baseline values with standard deviation (SD) and *p*-value (*p*) at baseline and day 19.

|  |  | **Baseline** | | | |  | **Day 19** | | | |
| --- | --- | --- | --- | --- | --- | --- | --- | --- | --- | --- |
|  |  | Mean (SD) | *p* | | Percent |  | Mean (SD) | *p* | Percent from baseline (SD) | *p* |
| EPO | MRH | 894 (322) | .63 | | 100 |  | 741 (250) | .12 | 90.3 (42.4) | **.005** |
|  | Normoxia | 996 (1262) |  |  | 100 |  | 1185 (1717) |  | 115 (28.9) |  |
| BDNF | MRH | 71275 (17472) | .81 | | 100 |  | 71496 (19281) | .60 | 105 (40.7) | .62 |
|  | Normoxia | 70406 (13287) |  |  | 100 |  | 69274 (16742) |  | 101 (26.7) |  |
| Note: Significant p-values are marked in bold. | | | |  |  |  |  |  |  |  |

**Table S6.** Intercept (β), confidence interval (CI), standard error and *p*-values for predictors of SpO2, ESQ, hemoglobin, thrombocytes, EPO, and cognitive learning.

| **Dependent variable** | **Predictor** | **β** | **95% CI** | | **Std. Error** | ***p*** |
| --- | --- | --- | --- | --- | --- | --- |
|  |  |  | **Lower** | **Upper** |  |  |
| **SPO2** |  |  |  |  |  |  |
|  | Time | 0.03 | -0.01 | 0.06 | 0.02 | .078 |
|  | Group | -0.05 | -0.19 | 1.08 | 0.60 | .931 |
|  | Age | -0.03 | -0.07 | 0.01 | 0.02 | .216 |
|  | Sex | 1.16 | 0.38 | 1.94 | 0.42 | **.007** |
|  | Day 1 SPO2 | 0.70 | 0.55 | 0.84 | 0.07 | **<.001** |
|  | Baseline HDRS17 | 0.02 | -0.09 | 0.12 | 0.06 | .735 |
|  | Verbal IQ | 0.03 | -0.05 | 0.10 | 0.04 | .468 |
|  | No. treatment days | 0.40 | 0.03 | 0.76 | 0.19 | **.044** |
| **ESQ** |  |  |  |  |  |  |
|  | Time | -0.06 | -0.14 | -0.04 | 0.03 | **.001** |
|  | Group | 3.59 | 1.20 | 5.98 | 1.27 | **.006** |
|  | Age | -0.12 | -0.21 | -0.03 | 0.05 | **.018** |
|  | Sex | -0.48 | -2.14 | 1.17 | 0.89 | .588 |
|  | Day 1 SPO2 | -0.13 | -0.42 | 0.16 | 0.16 | .418 |
|  | Baseline HDRS17 | -0.08 | -0.30 | 0.14 | 0.12 | .502 |
|  | Verbal IQ | -0.004 | -0.16 | 0.15 | 0.08 | .959 |
|  | No. treatment days | -0.85 | -1.61 | -0.08 | 0.41 | **.042** |
| **Hemoglobin** |  |  |  |  |  |  |
|  | Time – Day 19 | 3.83 | 2.36 | 5.31 | 0.75 | **<.001** |
|  | Group | -0.08 | -2.54 | 2.39 | 1.32 | .953 |
|  | Age | -0.02 | -0.11 | 0.08 | 0.05 | .755 |
|  | Sex | 0.07 | -1.60 | 1.74 | 0.89 | .938 |
|  | Day 1 SPO2 | ~~-~~0.16 | -0.45 | 0.129 | 0.16 | .304 |
|  | Baseline HDRS17 | 0.08 | -0.17 | 0.33 | 0.13 | .545 |
|  | Verbal IQ | 0.08 | -0.07 | 0.23 | 0.08 | .322 |
|  | No. treatment days | 0.03 | -0.74 | 0.79 | 0.41 | .948 |
| **Thrombocytes** |  |  |  |  |  |  |
|  | Time – Day 19 | 9.557 | 5.96 | 13.16 | 1.84 | **<.001** |
|  | Group | 1.23 | -5.71 | 8.20 | 3.72 | .742 |
|  | Age | -0.10 | -0.36 | 0.16 | 0.14 | .494 |
|  | Sex | -1.84 | -6.57 | 2.90 | 2.53 | .470 |
|  | Day 1 SPO2 | -0.47 | -1.30 | 0.36 | 0.44 | .294 |
|  | Baseline HDRS17 | -0.22 | -0.92 | 0.47 | 0.37 | .554 |
|  | Verbal IQ | 0.49 | 0.05 | 0.92 | 0.23 | **.041** |
|  | No. treatment days | -0.48 | -2.66 | 1.70 | 1.17 | .682 |
| **EPO** |  |  |  |  |  |  |
|  | Time – Day 19 | -8.46 | -19.63 | 2.70 | 5.94 | .158 |
|  | Group | -1.45 | -20.00 | 17.11 | 9.87 | .884 |
|  | Age | 0.34 | -0.39 | 1.07 | 0.39 | .386 |
|  | Sex | 5.35 | -7.01 | 17.71 | 6.57 | .418 |
|  | Day 1 SPO2 | 1.29 | -0.73 | 3.31 | 1.08 | .234 |
|  | Baseline HDRS17 | -0.46 | -2.31 | 1.39 | 0.98 | .639 |
|  | Verbal IQ | 0.31 | -0.82 | 1.44 | 0.60 | .609 |
|  | No. treatment days | -3.93 | -9.55 | 1.69 | 2.99 | .192 |
| **Learning** |  |  |  |  |  |  |
|  | Time | 0.52 | 0.48 | 0.56 | 0.02 | **<.001** |
|  | Group | -0.09 | -0.56 | 0.38 | 0.26 | .727 |
|  | Age | -0.01 | -0.02 | 0.01 | 0.01 | .550 |
|  | Sex | ~~-~~0.11 | -0.54 | 0.31 | 0.23 | .619 |
|  | Day 1 SPO2 | -0.01 | -0.07 | 0.06 | 0.04 | .883 |
|  | Baseline HDRS17 | 0.05 | 0.004 | 0.09 | 0.02 | .054 |
|  | Verbal IQ | -0.01 | -0.04 | 0.02 | 0.02 | .545 |
|  | No. treatment days | 0.15 | -0.03 | 0.33 | 0.10 | .146 |
| Notes: Significant *p*-values are marked in bold. | | | | | | |

**Figure S1.** Hematology parameters (percent change from baseline) at the time points day 0, day 8, and day 19, with error bars (standard errors). Blue represents moderate repeated hypoxia (MRH). Red represents EPO group.

**Figure S2.** Serum EPO and serum BDNF (percent change from baseline) at the time points day 1 and day 19, with error bars (standard errors). Blue represents moderate repeated hypoxia (MRH). Green represents normoxia group.

**References**

1	Macoveanu J, Petersen JZ, Mariegaard J, Jespersen AE, Cramer K, Bruun CF *et al.* Effects of erythropoietin on cognitive impairment and prefrontal cortex activity across affective disorders: A randomized, double-blinded, placebo-controlled trial. *J Psychopharmacol* 2024; **38**: 362–374.
